# The rice enhancer of zeste [E(z)] genes *SDG711* and *SDG718* are respectively involved in long day and short day signaling to mediate the accurate photoperiod control of flowering time

**DOI:** 10.3389/fpls.2014.00591

**Published:** 2014-10-31

**Authors:** Xiaoyun Liu, Chao Zhou, Yu Zhao, Shaoli Zhou, Wentao Wang, Dao-Xiu Zhou

**Affiliations:** ^1^National Key Laboratory of Crop Genetic Improvement, Huazhong Agricultural UniversityWuhan, China; ^2^Institut de Biologie des Plantes, UMR8618, Université Paris-Sud 11Orsay, France

**Keywords:** *Oryza sativa*, heading date, epigenetics, epigenomics, histone methylation, PRC2, *OsCLF*, *OsiEZ1*

## Abstract

Recent advances in rice flowering studies have shown that the accurate control of flowering by photoperiod is regulated by key mechanisms that involve the regulation of flowering genes including *Heading date1 (Hd1)*, *Early hd1 (Ehd1)*, *Hd3a,* and *RFT1*. The chromatin mechanism involved in the regulation of rice flowering genes is presently not well known. Here we show that the rice enhancer of zeste [E(z)] genes *SDG711* and *SDG718,* which encode the polycomb repressive complex2 (PRC2) key subunit that is required for trimethylation of histone H3 lysine 27 (H3K27me3), are respectively, involved in long day (LD) and short day (SD) regulation of key flowering genes. The expression of *SDG711* and *SDG718* is induced by LD and SD, respectively. Over-expression and down-regulation of *SDG711* respectively, repressed and promoted flowering in LD, but had no effect in SD. By contrast, down-regulation of *SDG718* had no effect in LD but delayed flowering in SD. *SDG711* and *SDG718* repressed *OsLF* (a repressor of *Hd1*) respectively in LD and SD, leading to a higher expression of *Hd1* thus late flowering in LD and early flowering in SD. *SDG711* was also found to be involved in the repression of *Ehd1* in LD. SDG711 was shown to directly target to *OsLF* and *Ehd1* loci to mediate H3K27me3 and gene repression. The function of the rice E(z) genes in LD repression and SD promotion of flowering suggests that PRC2-mediated epigenetic repression of gene expression is involved in the accurate photoperiod control of rice flowering.

## INTRODUCTION

The control of flowering time is a critical step for successful grain production in rice. Day length is a key factor controlling rice flowering. Most rice cultivars recognize 13.5 h light/10.5 h dark as a critical photoperiod to separate long day (LD) from short day (SD) periods, day length shorter than 13.5 h will greatly induce rice flowering (Itoh et al., 2010; Tsuji et al., 2013). Despite some genes are shared between *Arabidopsis* and rice flowering regulatory pathways, there are considerable differences between the regulation of flowering of both species such as absence of the vernalization pathway in rice (Shrestha et al., 2014). *Heading date1 (Hd1)*, the rice ortholog of *Arabidopsis CONSTANS* (*CO*), has a dual role in flowering time control. It promotes flowering at SD and represses flowering in LD (Yano et al., 2000). (*Hd3a* is a rice ortholog of the *Arabidopsis* florigen gene *FLOWERING LOCUS T* (*FT*; Yano et al., 2000; Kojima et al., 2002; Tamaki et al., 2007). Hd1 activates *Hd3a* in SD, but in LD Hd1 is converted by phytochrome B to a repressor of *Hd3a* (Yano et al., 2000; Hayama and Coupland, 2004). *Hd1* expression is controlled by *Oryza sativa* GIGANTEA (OsGI), a key factor of circadian rhythms in rice flowering control (Yano et al., 2000; Hayama et al., 2003). There is a second, *Hd1*-independent, photoperiod inductive pathway in rice. *Early hd1* (*Ehd1*), a B-type response regulator that is activated by a SD flowering promoter (i.e., *OsMADS51*), activates the expression of *Hd3a*, *RFT1* (another Rice *FT* gene), and *OsMADS14,* and mainly confers SD-dependent flowering promotion (Doi et al., 2004; Kim et al., 2007). In LD, *Ehd1* is activated by *Ehd2/OsId1/RID1* (*Rice Indeterminate1*) and *OsMADS50*, but repressed by *Ghd7* (*Grain number, plant height and heading date7*; Lee et al., 2004; Matsubara et al., 2008; Park et al., 2008; Wu et al., 2008; Xue et al., 2008; Itoh et al., 2010; Tsuji et al., 2013). *Ehd1* activates the expression of *Hd3a* and *RFT1* which activates *MADS14* and *MADS15* (Komiya et al., 2009). The tight control of expression of the flowering promoter *Ehd1* and the flowering repressor *Ghd7* allows to measure the slight differences in day lengths to control *Hd3a* and *RFT1* transcription with a critical day length threshold (Itoh et al., 2010; Tsuji et al., 2013). Unlike *Hd1* that is conserved with *Arabidopsis CO*, *Ehd1,* and *Ghd7* are evolutionarily acquired rice-specific genes. Therefore, the control of florigen expression is regulated by key mechanisms that involve the regulation of *Hd1* expression, the conversion of Hd1 function on *Hd3a* expression, and modulation of *Ehd1/Ghd7* expression.

Recent studies have established a close relationship of epigenetic regulation with flowering. For instance, during the process of vernalization in *Arabidopsis*, polycomb repressive complex 2 (PRC2)-mediated trimethylation of histone H3 lysine 27 (H3K27me3) represses the expression of *Flowering Locus C* (*FLC*) to stimulate flowering (Sung and Amasino, 2004; De Lucia et al., 2008). In rice, SDG724 mediates H3K36me2/3 deposition at *OsMADS50* and *RFT1*, promoting flowering and establishing a difference of functionality between paralogs *RFT1* and *Hd3a* under LD or SD conditions (Sun et al., 2012). SDG725 also mediates H3K36me2/3 deposition at *Ehd2*, *Ehd3*, *OsMADS50*, *Hd3a,* and *RFT1*, promoting flowering under LD or SD conditions (Sui et al., 2013). The PRC2 complex was first discovered in *Drosophila*, which has four core proteins: ENHANCER OF ZESTE [E(z)], SUPPRESSOR OF ZESTE 12 [Su(z)12], EXTRA SEX COMBS (ESC) and P55 (Schuettengruber and Cavalli, 2009). The E(z) protein has the H3K27 methyltransferase activity (Cao et al., 2002; Czermin et al., 2002). *Arabidopsis* contains three E(z) genes [*CURLY LEAF* (*CLF*), *SWINGER* (*SWN*), and *MEDEA* (*MEA*)], three Su(z)12 genes [*FERTILIZATION INDEPENDENT SEED 2* (*FIS2*), *VERNALIZATION 2* (*VRN2*) and *EMBRYONIC FLOWER 2* (*EMF2*)], and one ESC gene [*FERTILIZATION INDEPENDENT ENDOSPERM* (*FIE*)] (Luo et al., 1999; Ohad et al., 1999; Gendall et al., 2001; Yoshida et al., 2001; Hennig et al., 2003), which form three PRC2-like complexes. The FIS complex, which contains MEA/SWN, FIS2, FIE, and MSI1 (Multicopy Suppressor of IRA1), functions during gametogenesis and seed development. The others are involved in flowering control: the EMF complex that is comprised of CLF/SWN, EMF2, FIE, and MSI1 and is involved in the suppression of early flowering and the VRN complex that plays critical roles in the vernalization pathway by maintaining the high level of H3K27me3 on the *FLC* locus after vernalization (Hennig and Derkacheva, 2009). The VRN complex is associated with VERNALIZATION INSENSITIVE 3 (VIN3, a PHD-domain containing protein) and VIN3-like proteins to form PHD-VRN PRC2 complexes (Wood et al., 2006; De Lucia et al., 2008). The VIN3 protein enhances H3K27me3 throughout the target loci to a level sufficient for stable silencing.

The rice genome contains two genes for E(z) (*OsiEZ1* and *OsCLF*), Su(Z)12 (*OsEMF2a* and *OsEMF2b*) and ESC (*OsFIE1* and *OsFIE2;* Luo et al., 2009). No morphological changes are observed in *osclf* and *osfie1* mutants, while *osfie2* and *osemf2b* mutants display earlier flowering at LD and abnormal flower organs (Luo et al., 2009). Recent results have shown that rice VIN3-like proteins OsVIL2, OsVIL3, or RICE LEAF INCLINATION 2 (LC2, hereafter referred to as LC2) promote rice flowering through the photoperiod pathway (Wang et al., 2013; Yang et al., 2013). These results suggest that PRC2 and PRC2-associated genes are involved in photoperiod regulation of flowering in rice. However, how PRC2-mediated gene repression is involved in accurate photoperiod control of rice flowering is not clear.

In this work we show that the two rice E(z) genes, *OsCLF* (or *SDG711,* Os06g16390, here after referred to as *SDG711*) and *OsiEZ1* (or *SDG718,* Os03g19480, here after referred to as *SDG718*), displayed distinct function in photoperiod regulation of flowering in rice. *SDG718* is induced in SD and represses *OsLF*, a repressor of Hd1 (Zhao et al., 2011), leading to a higher expression of *Hd1* (that activates *Hd3a* in SD) and thus early flowering. *SDG711* is induced in LD and represses *OsLF*, *Ehd1*, and other flowering-promoting genes leading to late flowering in LD. The data suggested that the two E(z) genes are involved respectively, in LD and SD signals to differentially control key flowering genes expression and contribute to the accurate photoperiod control of flowering time in rice.

## MATERIALS AND METHODS

### PLANT MATERIALS AND GROWING CONDITIONS

Rice cultivar (*Oryza sativa* spp. *japonica*) “Zhonghua 11” (ZH11) and “DongJin” (DJ) were used for genetic transformation in this study. T-DNA insertion line of *SDG711* (3A-60654.R) was obtained from the Postech rice mutant database (http://signal.salk.edu/cgi-bin/RiceGE). Insertion was confirmed by PCR using the specific primers F and R and a T-DNA left side primer RB2. The primers used for genotyping and real-time PCR analysis are listed in Table S1. The rice plants were grown either in a paddy field in summer in Wuhan (day length >13.5 h) or in controlled growth chambers for 6 week-old under SD (10 h light at 30°C/14 h dark at 25°C) or 8 week-old LDs (14 h light at 30°C/10 h dark at 25°C) conditions as described previously (Yang et al., 2013).

### EXPRESSION ANALYSIS BY NORTHERN BLOTS AND RT-PCR

Total RNA was isolated from rice callus, stems, leaves, flag leaves, shoots, panicles, endosperm, and roots using TRIzol reagent (Invitrogen). Three μg of total RNA were reverse-transcribed in a reaction of 20 μl by using DNase I and SuperScript III (Invitrogen) according to the manufacturer’s instruction to obtain cDNA. For northern blotting analysis, fifteen micrograms of total RNA extracted from field-grown rice leaves was separated in 1.2% (w/v) formamide-denaturing agarose gels, then transferred to nylon membranes. Gene-specific probes were labeled with ^32^P-dCTP using the Random Primer kit (Invitrogen) and hybridized to the RNA blots. The probe of *SDG711* was amplified from *SDG711* cDNA using primers Insitu-SDG711-F and Insitu-SDG711-R (Table S1), resulting in a fragment of 505 bp of the cDNA.

Real-time PCR was performed in an optical 96-well plate that included SYBR Premix EX Taq and 0.5 μl of Rox Reference Dye II (Takara), 1 μl of the reverse transcription reaction, and 0.25 μM of each gene-specific primer in a final volume of 25 μl on a PRISM 7500 PCR instrument (Applied Biosystems). The reactions were performed at 95°C for 10 s, 45 cycles of 95°C for 5 s, and 60°C for 40 s. Disassociation curve analysis was performed as follows: 95°C for 15 s, 60°C for 20 s, and 95°C for 15 s. Data were collected using the ABI PRISM 7500 sequence detection system following the instruction manual. The relative expression levels were analyzed using the 2–ΔΔCT method (Livak and Schmittgen, 2001). The rice *ACTIN1* gene was used as the internal control. Accession numbers of genes analyzed in this study: *SDG711*: LOC_Os06g16390; *SDG718*: LOC_Os03g19480; *OsLF*: LOC_Os05g46370; *Ehd1*: LOC_Os10g32600; *RFT1*: LOC_Os06g06300; *Hd1*: LOC_Os06g16370; *Hd3a*: LOC_Os06g06320; *RID1*: LOC_Os10g28330; *OsGI*: LOC_Os01g08700; *Ghd7*: LOC_Os07g15770; *OsMADS14*: LOC_Os03g54160; *OsMADS15*: LOC_Os07g01820; *OsMADS50*: LOC_Os03g03070; *OsMADS51*: LOC_Os01g69850. The primers for real-time PCR are listed in Table S1.

### VECTOR CONSTRUCTION AND PLANT TRANSFORMATION

For over-expression (OX) vector, the *SDG711* full-length cDNA was amplified from DJ leaf mRNA using primer set OXSDG711-F and OXSDG711-R, then inserted into the OX vector pU1301 under the control of the maize ubiquitin gene promoter within *Kpn*I sites (Sun and Zhou, 2008). For RNAi vectors, gene-specific sequences of *SDG711* and *SDG718*, spanning from 2352 bp to 2860 bp relative to the translation start site and from 918 bp to 1599 bp relative to the translation start site, respectively, were amplified from cDNA using primer sets RiSDG711-F/RiSDG711-R and RiSDG718-F/RiSDG718-R, then inserted into the RNAi vector pDS1301 (Huang et al., 2007). Sequence amplified using the primers sets are listed in Table S1. The constructs were transformed into DJ (SDG711 OX and RNAi) and ZH11 (*SDG718* RNAi) plants by *Agrobacterium tumefaciens* (strain *EHA105) –* mediated transformation as previously described (Huang et al., 2007).

### WESTERN BLOTTING ANALYSIS

For Western blot analysis, histone-enriched fractions were extracted from wild type (WT), mutant, and transgenic leaves as described previously (Huang et al., 2007). Antibodies used in Western blot are: anti-H3K27me3 (07-449, Millipore), anti-H3K27me2 (ab24684, Abcam), anti-H3K27me1 (ab113671, Abcam), anti-H3 (ab1791, Abcam), anti-H3K4me3 (07-473, Millipore), anti-H3K4me2 (07-430, Millipore), anti-H3K4me1 (07-436, Millipore), anti-H3K9me3 (ab8898, Abcam), anti-H3K9me2 (07-441, Millipore), anti-H3K9me1 (ab9045, Abcam) H3K36me1 (ab9048, Abcam), anti-H3K36me2 (ab9049, Abcam), and anti-H3K36me3 (ab9050, Abcam). Anti-SDG711 was prepared by immunizing rabbits with SDG711 protein produced in *Escherichia coli* (in pET-28a vector) and purified with His-tag protein purification beads (V8550, GE Healthcare). The anti-serum was affinity-purified with protein-A agarose beads purchased from Millipore (16-157).

### CHROMATIN IMMUNOPRECIPITATION (ChIP)

The chromatin immunoprecipitation (ChIP) experiment was performed as described (Huang et al., 2007). Rice leaves (0.8–1.0 g fresh weight) were harvested at the end of the dark period of 8 week-old LD grown and 6 week-old SD grown plants and crosslinked in 1% formaldehyde under vacuum. Chromatin was extracted and fragmented to 200–750 bp by sonication, and ChIP was performed using the following antibodies: H3K27me3 (07-449, Millipore), H3K4me3 (07-473, Millipore), and Anti-SDG711. The precipitated and input DNA samples were then analyzed by real-time PCR with gene-specific primers listed in Table S1. All assays were performed at least three times from two biological replicates.

## RESULTS

### THE RICE E(z) HOMOLOGOUS GENES DISPLAY DISTINCT PHOTOPERIODIC EXPRESSION PATTERNS

**FIGURE 1:**
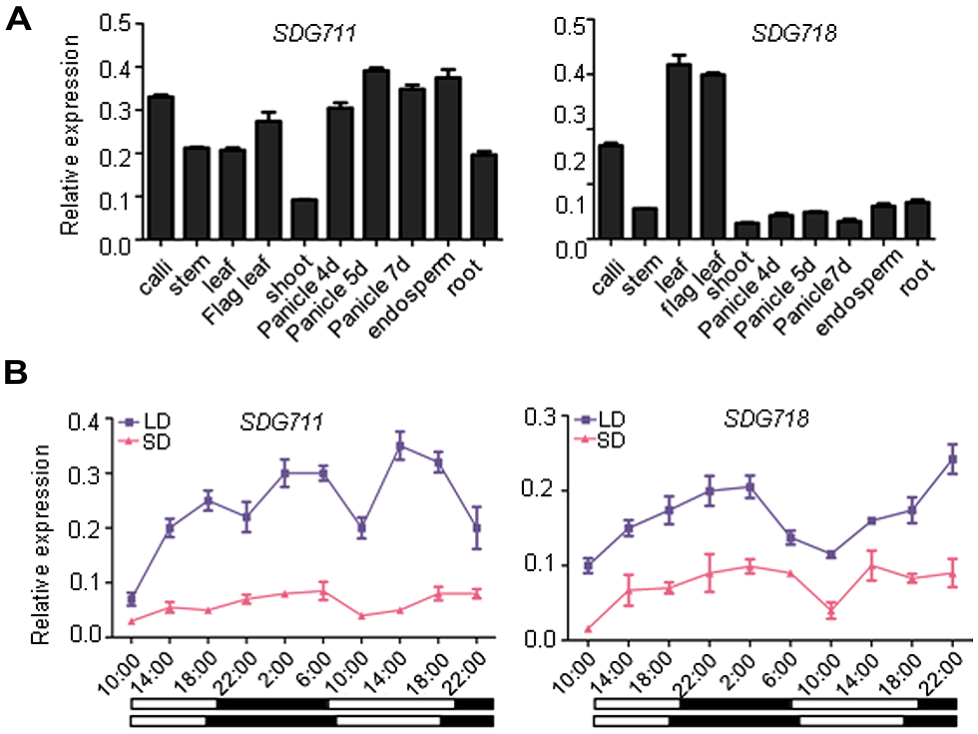
**Expression profiles of *SDG711* and *SDG718*. (A)** Real-time PCR detection of transcript levels of the genes in indicated organs or developmental stages. **(B)** transcript levels of the two genes in young leaves harvested at intervals of 4 h during a 36 h period from plants grown under long day (LD) or short day (SD) conditions. Dark periods are indicated by black bars. Bars = mean ± SD from two biological repeats. Values are shown as relative to *ACTIN* transcript levels.

Phylogenetic analysis has previously shown that rice E(z) homologous genes, *SDG711* and *SDG718*, are closely related to *Arabidopsis CLF* and *SWN*, respectively (Luo et al., 2009; Figure S1). The two rice genes share 51% amino acid sequence identity, with much higher conservations in the catalytic SET domain and the protein interaction domains (SAND, Cys-rich). Analysis of expression of the genes by real-time PCR indicated that *SDG711* was widely expressed in different tissues/organs, whereas *SDG718* was more expressed in leaves than other tested tissues/organs (**Figure 1A**). Because *OsEMF2b* and PRC2-interacting PHD domain protein genes are involved in photoperiod regulation of flowering time in rice (Luo et al., 2009; Wang et al., 2013; Yang et al., 2013), we studied whether the rice E(z) genes were photoperiod-responsive. We analyzed mRNA isolated from leaves of 6 week-old SD (10 h light/14 h dark)- and 8 week-old LD (14 h light/10 h dark)- grown plant leaves harvested at intervals of 4 h during a 36 h period. The analysis revealed that *SDG711* mRNA levels were higher in LD than in SD, whereas that of *SDG718* were higher in SD than LD (**Figure 1B**).

### *SDG711* IS INVOLVED IN LD REPRESSION OF RICE FLOWERING

**FIGURE 2:**
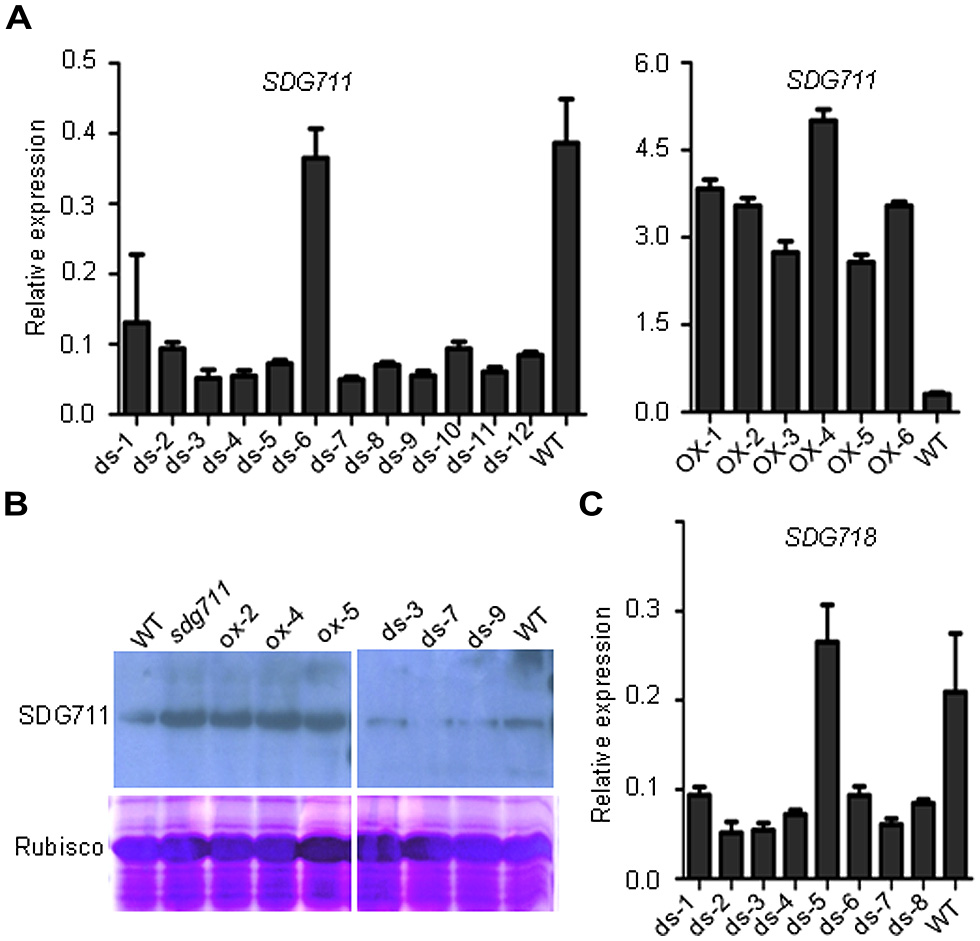
**Characterization of *SDG711* and *SDG718* transgenic plants. (A)** Detection of *SDG711* mRNA levels in wild type (WT) and 12 independent RNAi (ds; left), and six over-expression (OX) lines (right).**(B)** Detection of SDG711 protein levels in WT, three OX and three RNAi lines, and the gain-of-function mutant *(sdg711*). **(C)** Detection of *SDG718* mRNA levels in WT and eight independent RNAi lines. Y-axis: relative expression levels to *ACTIN* transcripts. Bars = means ± SD from three technical repeats.

**FIGURE 3:**
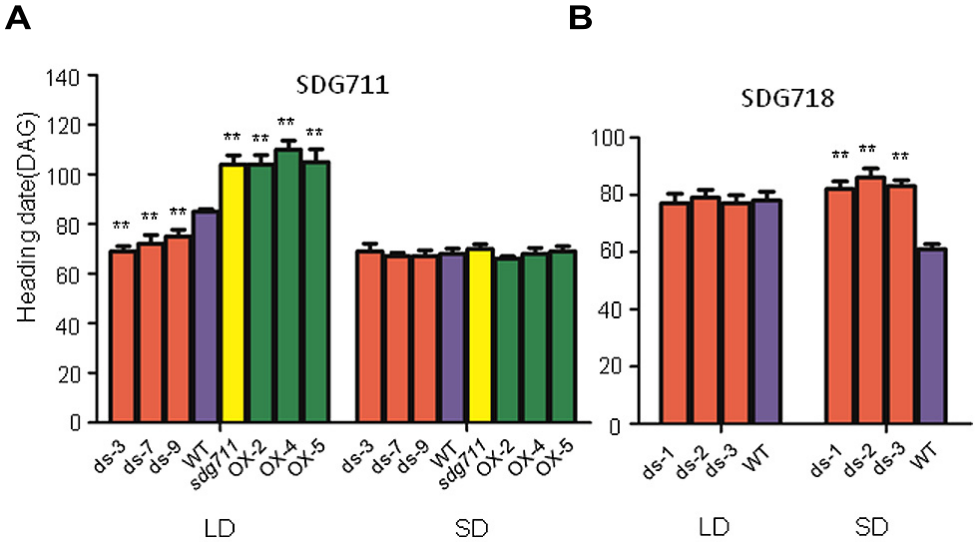
**Flowering time of *SDG711* and *SDG718* transgenic plants. (A)** Heading date (DAG: days after germination) of 3 *SDG711* RNAi (ds) lines, three OX lines and the gain-of-function mutant (*sdg711*) compared to WT in LD and SD conditions. **(B)** Heading date of three *SDG718* RNAi (ds) lines compared to WT in LD and SD conditions. Twenty plants per genotype were surveyed. Significance of differences between WT and transgenic plants are indicated by (^∗∗^*p* < 0.01, Student’s *t*-test).

To study the function of *SDG711*, we produced transgenic rice plants in DJ which contains functional *Hd1* (Naranjo et al., 2014) to knockdown the gene by RNAi and to over-express the gene by using the maize ubiquitin gene promoter (Sun and Zhou, 2008). Analysis of the transgenic plants revealed several lines with reduced expression and six lines with increased expression of the gene (**Figure 2A**). To check the protein level of SDG711, we performed Western blot analysis of protein extracts from the leaves of the WT and transgenic plants grown in LD by using antibodies generated against *E. coli*-produced SDG711 protein. The analysis confirmed the OX and RNAi of the gene in the transgenic lines (**Figure 2B**). Three single copy T3 homozygous lines (offspring of single insertion T2 lines that did not segregate transgene-negative individuals) per transgene were selected for phenotypic analysis. During vegetative growth the transgenic plants did not display any visible morphological defects but exhibited altered HD (or flowering time) compared to the WT plants. In LD (14 h light/10 h dark), the HD of the OX plants was largely delayed (20–25 days, *p* < 0.001, student’s *t*-test), while that of the RNAi plants was significantly earlier (10–16 days, *p* < 0.001, student’s *t*-test) than WT (**Figure 3**). However, in SD (10 h light/14 h dark) the HD of *SDG711* OX and RNAi plants was not significantly different from WT (**Figure 3**). These data suggested that *SDG711* may have a function to repress flowering in LD. In addition to the effect on flowering time, the OX plants produced a higher number of stamens (Figure S2). The pollen viability was reduced in both the OX and RNAi plants of *SDG711* (Figure S2). These observations suggested that *SDG711* might play a role in fertility. To confirm the transgenic results, we characterized a T-DNA mutant that had the insertion located in the 5′-UTR of the gene (Figures S3A,B). One single insertion was identified by Southern blotting (Figure S3C). Real-time PCR revealed a higher level of transcripts of the gene (Figure S3D). Northern blotting experiments revealed that the increased transcripts were about the same size as in WT (Figure S3E). The mutant displayed the same phenotype as that of the OX plants (**Figure 3**). After three backcrosses the phenotype co-segregated with the presence of the insertion, indicating that the mutation was a gain-of-function mutation.

To study the effect of *SDG711* OX or RNAi on histone methylation, we performed Western blotting analysis of histones isolated from leaves of the gain-of-function mutant, an RNAi and an OX line. The analysis revealed that compared to WT the levels of H3K27me3 were lower in the RNAi line, but higher in the gain-of-function mutant and OX plants (Figure S4), suggesting that *SDG711* was required for the overall H3K27me2/3 in rice.

**FIGURE 4:**
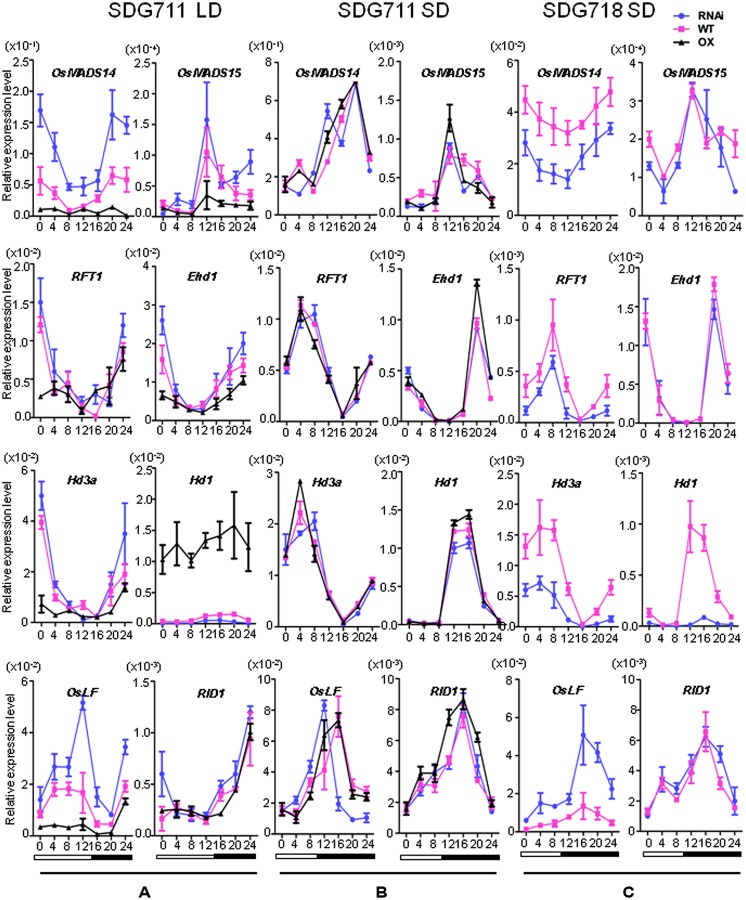
**Expression levels of key flowering regulatory genes in mature leaves of *SDG711* and *SDG718* transgenic plants compared to WT. (A)** Key flowering genes expression in *SDG711* transgenic plants compared to WT in LD. **(B)** Key flowering genes expression in *SDG711* transgenic plants compared to WT in SD. **(C)** Key flowering genes expression in *SDG718* RNAi plants compared to WT in SD. Leaves of transgenic plants were harvested from plants of 3 T3 lines grown for 8 week-old under LDs and 6 week-old under SDs. Bars = mean ± SD from three biological repeats.

The flowering time phenotype suggested that *SDG711* had a function to suppress flowering in LD while without a clear effect in SD. We therefore analyzed the expression of flowering regulatory genes in both pathways. mRNA were isolated from 8 week-old LD or 6 week-old SD plant leaves at intervals of 4 h during a 24 h period. In LD, the mRNA levels of LD flowering activators including *Ehd1*, *Hd3a*, *MADS14*, and *MADS15* were clearly decreased in the OX, but increased in the RNAi plants compared to WT (**Figure 4A**). By contrast, the transcript levels of the LD flowering repressor *Ghd7* were not clearly altered in the transgenic plants (Figure S5). The expression of *RID1* and *OsMADS50* also appeared unchanged in the transgenic plants (**Figure 4A**; Figure S5). Interestingly, *Hd1* that acts as a repressor of *Hd3a* in LD was highly induced by *SDG711* OX and repressed by *SDG711* RNAi (**Figure 4A**), while the expression level of *OsGI* that activates *Hd1* was not changed (Figure S5). Because E(z) proteins are supposed to be transcriptional repressors, the activation of *Hd1* in *SDG711* OX plants might be due to an indirect effect. Recent data have shown that OsLF, a bHLH protein that directly represses *Hd1* (Wang et al., 2013). Examination of *OsLF* transcripts in the transgenic plants indicated that *OsLF* was repressed by *SDG711* OX, but activated by *SDG711* RNAi in LD (**Figure 4A**). The expression of flowering regulatory genes supported the flowering time phenotype observed in the transgenic plants (**Figure 3**). Analysis of the gain-of-function mutant confirmed the above observed effects of *SDG711* OX on flowering time gene expression (Figure S6). In SD, the expression levels of the flowering genes were not clearly affected by *SDG711* OX or RNAi, except the peak expression of *OsLF* in the RNAi plants was about 4 h earlier than the WT (**Figure 4B**). These results suggested that *SDG711* is mostly involved in LD repression of rice flowering time.

**FIGURE 5:**
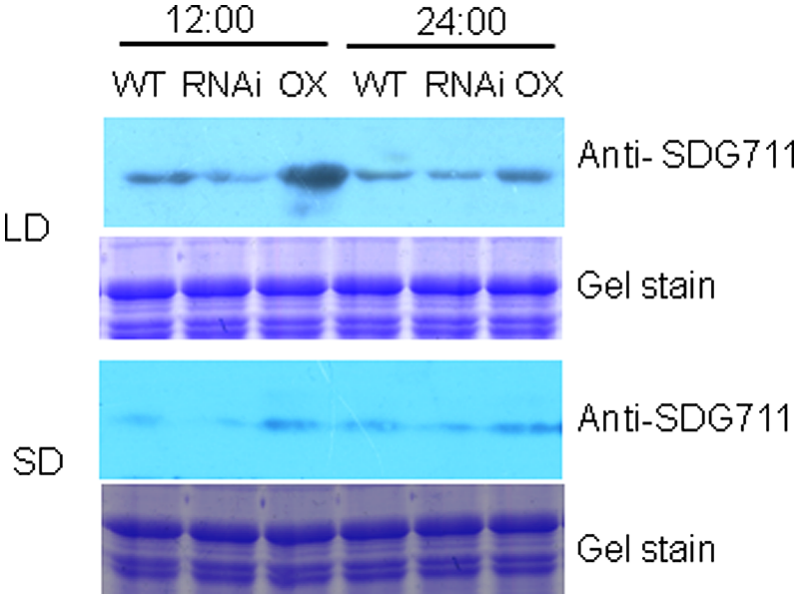
**SDG711 protein accumulation in LD - and SD- grown WT, *SDG711* RNAi and OX mature leaves of plants from 3 T3 lines harvested at noon (12:00) and mid night (24:00).** Protein extracts were resolved by SDS-PAGE and Coomassie stained before Western blotting with anti-SDG711.

To study whether SDG711 protein levels were differentially regulated in LD and SD, Western blotting analysis of protein extracts from WT, RNAi and OX plants grown in LD and SD harvested at 12:00 (mid day, after 7 h in light for LD condition and 5 h in light for SD condition) and 24:00 am (mid night, after 5 h in dark for LD condition and 7 h in dark for SD condition) was performed by using anti-SDG711. The results shown in **Figure 5** revealed that SDG711 protein levels were lower in SD compared in LD and that the high SDG711 level of the OX line was reduced in SD and in the dark, suggesting that the stability of SDG711 protein may be regulated by day length and light/dark conditions.

### *SDG718* PROMOTES FLOWERING IN SD

To study whether *SDG718* had a function in flowering time control, we obtained several RNAi lines in ZH11 variety (**Figure 2C**), which also has a functional Hd1 (Luan et al., 2009; Naranjo et al., 2014). These plants showed a similar HD as the WT in LD, but flowered later (>20 days) than WT in SD (**Figure 3B**). Analysis of flowering time gene expression revealed that *SDG718* RNAi clearly induced *OsLF*, but repressed the SD activators including *Hd1*, *Hd3a*, *OsMADS14,* and *RFT1* (**Figure 4C**), suggesting that *SDG718* played a role in promoting flowering in SD. However, the *SDG718* RNAi plants did not show any clear defect in flower organ and pollen viability (Figure S2).

### *SDG711* AND *SDG718* REGULATE H3K27me3 ON FLOWERING GENES

**FIGURE 6:**
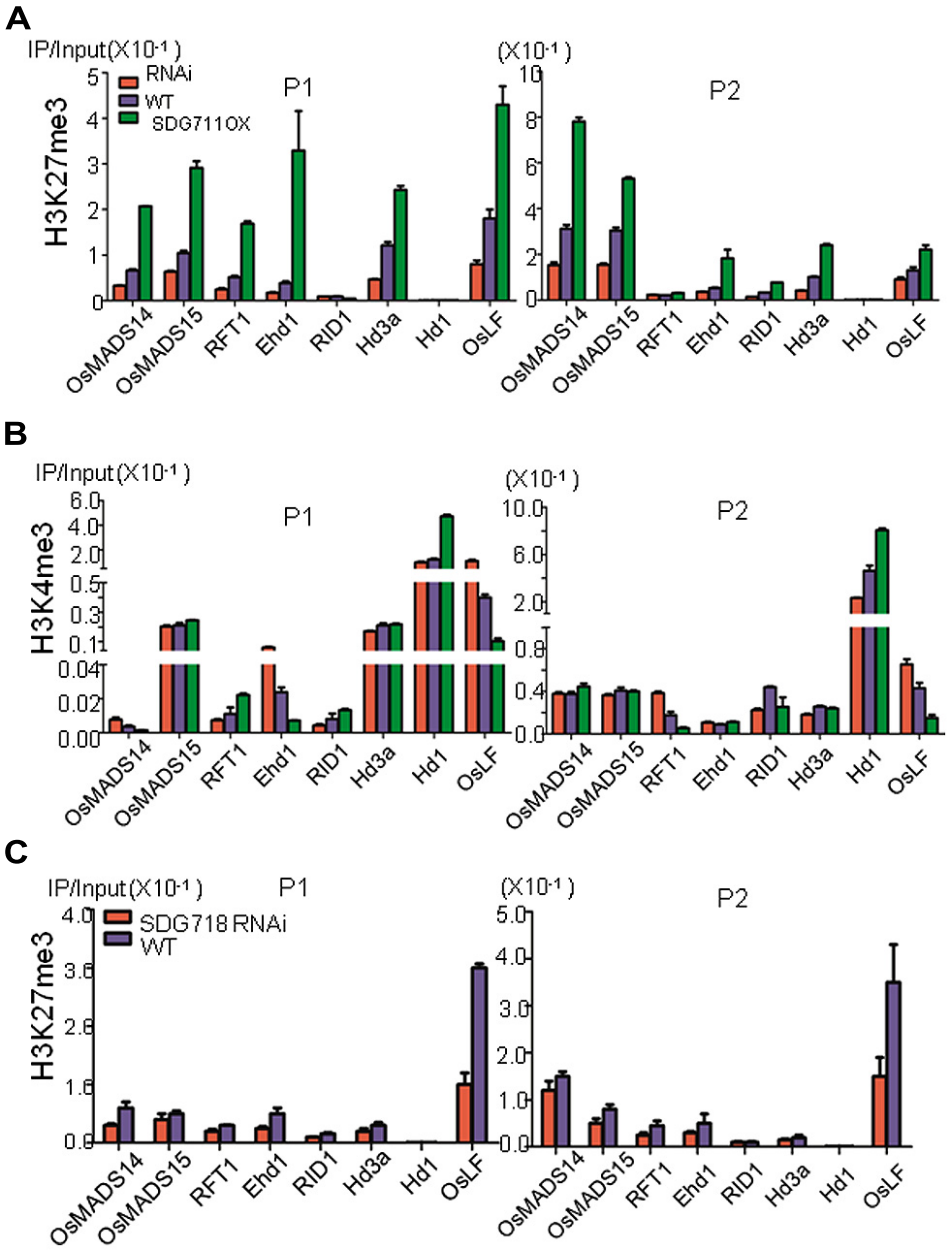
***SDG711* and *SDG718* function in H3K27me3 and H3K4me3 on key flowering genes. (A)** chromatin immunoprecipitation (ChIP) analysis of H3K27me3 on the indicated flowering genes in WT, *SDG711* RNAi and OX plants (mature leaves pooled from 3 T3 lines). **(B)** ChIP analysis of H3K4me3 on the indicated flowering genes in WT, *SDG711* RNAi and OX plants (pooled from 3 T3 lines). H3K27me3 and H3K4me3 enrichments on the 5′ end (P1) and the coding region (P2) of the flowering genes were detected by quantitative PCR. Three biological repeats were performed, one repetition is shown. One other biological replicate is shown in Figure S7. Bars = mean ± SD from three technical repeats. **(C)** ChIP analysis of H3K27me3 on the indicated flowering genes in WT and *SDG718* RNAi plants pooled from 3 T3 lines. Leaves of *SDG711* transgenic plants were harvested from 8 week-old LD plants grown, and leaves of *SDG718* transgenic plants were harvested from 6 week-old SD plants grown.

To study whether knockdown and OX of *SDG711* altered chromatin modification of flowering regulatory genes, we analyzed H3K27me3 on *RID1*, *Ehd1*, *RFT1*, *Hd3a*, *MADS14*, *MADS15*, *Hd1*, and *OsLF* in WT, OX and RNAi plants grown under LD conditions by ChIP assays. The 8 week-old plant leaves were harvested at the end of the darkness period under LD conditions. Because H3K27me3 is located in gene body with enrichment on the 5′ end of the gene in rice (Hu et al., 2012; Li et al., 2013), we analyzed the ChIP by real-time PCR using two primer sets, one corresponding to the 5′ region, the other to the gene body (**Figure 6**). Among these genes, *Hd1* appeared to be unmodified by H3K27me3 in either the 5′ region or the gene body, further supporting an indirect effect of *SDG711* on its expression. *RID* displayed a very low level of H3K27me3 (**Figure 6A**), consistent with the observation that the expression of the gene was not affected in the *SDG711* transgenic and the gain-of-function mutant plants (**Figure 4**; Figures S5 and S6). The remaining genes displayed H3K27me3 in the 5′ region and/or gene body, suggesting that regulation of these genes might involve PRC2 function. H3K27me3 on these genes (except the gene body of *RFT1* which displayed a relatively weak level of H3K27me3) was clearly reduced in the RNAi but increased in the OX plants (**Figure 6A**), which reversely correlated with their expression changes in the transgenic plants. These data suggested that SDG711-mediated H3K27me3 was involved in the regulation of the genes.

Because H3K27me3 is antagonistic to H3K4me3 on gene activity, we therefore analyzed whether alteration of H3K27me3 affected H3K4me3 on the flowering genes in the transgenic plants. The analysis revealed relatively higher levels of H3K4me3 in *Hd1*, *Hd3a*, *MADS15*, *Ehd1,* and *OsLF* than in *MADS14*, *RFT1,* and *RID* in WT plants (**Figure 6B**). H3K4me3 levels in *Hd1*, *MADS15,* and *Hd3a* were not affected by the *SDG711* transgenes except some increases on *Hd1* in the OX plants (**Figure 6B**). The increased H3K4me3 on *Hd1* may be due to increased expression of the gene in the OX plants, as H3K4me3 is thought to be associated with active genes (Hu et al., 2011). However, H3K4me3 on *Ehd1* and *OsLF* was increased in *SDG711* RNAi but decreased in *SDG711* OX plants (**Figure 6B**), which conversely correlated to that of H3K27me3 and suggested that *SDG711*-mediated H3K27me3 might affect H3K4me3 in the two loci, which may be linked to the repression of the genes. Analysis of *SDG718* RNAi plants also revealed a clear decrease of H3K27me3 in *OsLF* in SD (**Figure 6C**).

### SDG711 BINDS TO Ehd1 AND OsLF LOCI

**FIGURE 7:**
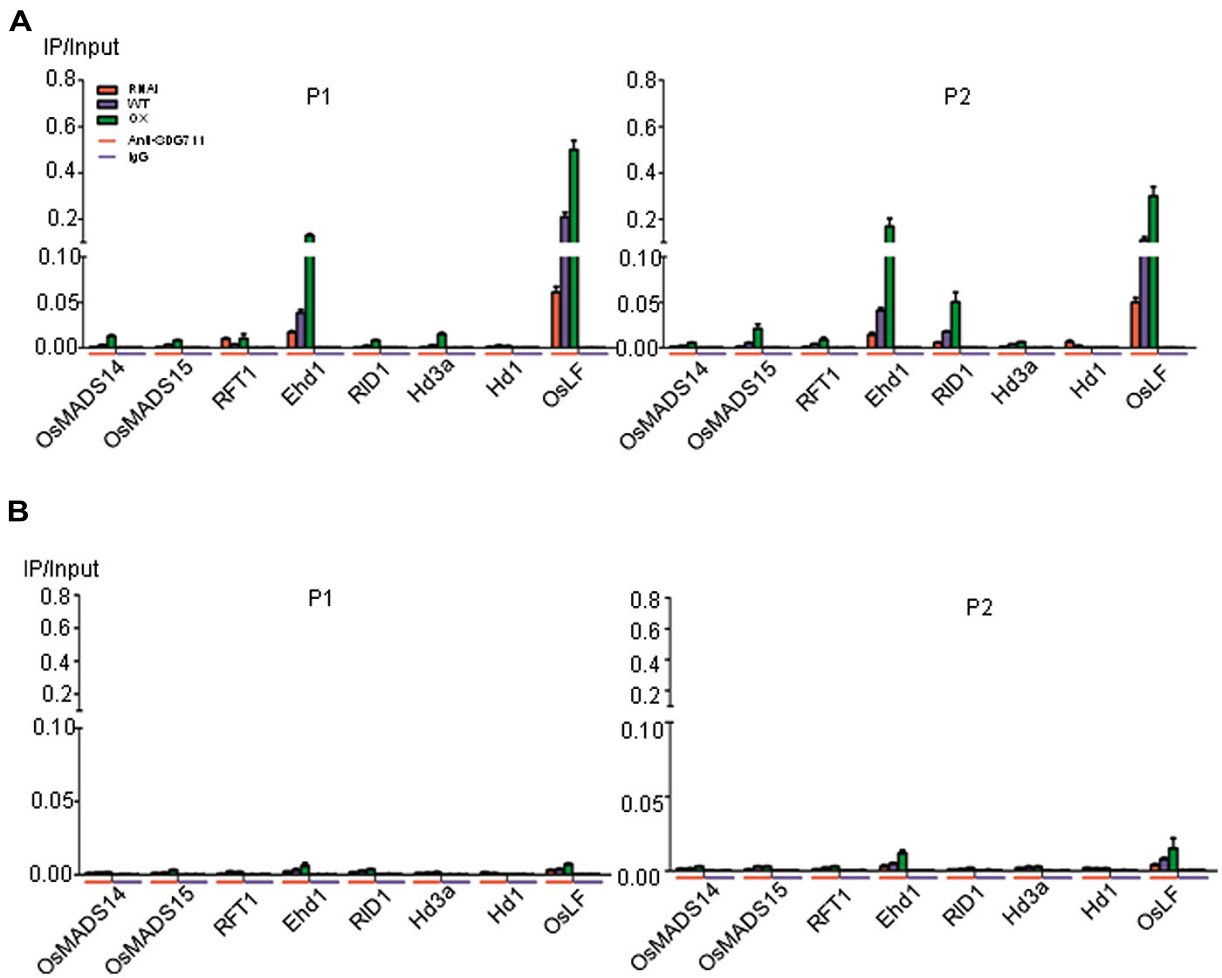
**Direct association of SDG711 protein with flowering genes. (A)** SDG711 protein enrichment on the indicated flowering genes in LD-grown WT, *SDG711* RNAi and OX seedlings tested by ChIP with anti-SDG711. Non-immunized rabbit serum (IgG) was used as control. Three biological repeats were performed, one repetition is shown. The two other repeats are shown in Figure S8. Bars = means ± SD from three technical repeats. **(B)** SDG711 protein enrichment on the indicated flowering genes in SD-grown plants. Leaves were harvested from plants grown for 8 week-old LD and 6 week-old SD plants grown.

To further assess the function of *SDG711* on the flowering gene regulation, we performed anti-SDG711 ChIP assays and analyzed by real-time PCR using the same primer sets as for the histone methylation ChIP. Non-immunized serum was used as control. The analysis revealed that in LD-grown WT plants SDG711 binding was clearly enriched in the 5′ end and the gene body of *Ehd1* and *OsLF* compared to the other tested genes (**Figure 7A**). However, some enrichment was also observed in the gene body of *RID1*. The SDG711 enrichment on the three genes was sensibly reduced in RNAi and clearly enhanced in the OX plants (**Figure 7A**), suggesting that SDG711 may directly target to the genes in LD. In SD-grown plants, the enrichment of SDG711 on the three genes was much weaker than that in SD (**Figure 7B**), which was consistent with lower accumulation of SDG711 in SD (**Figure 5**) and suggested that SDG711 enrichment in the flowering genes was regulated by day length.

## DISCUSSION

**FIGURE 8:**
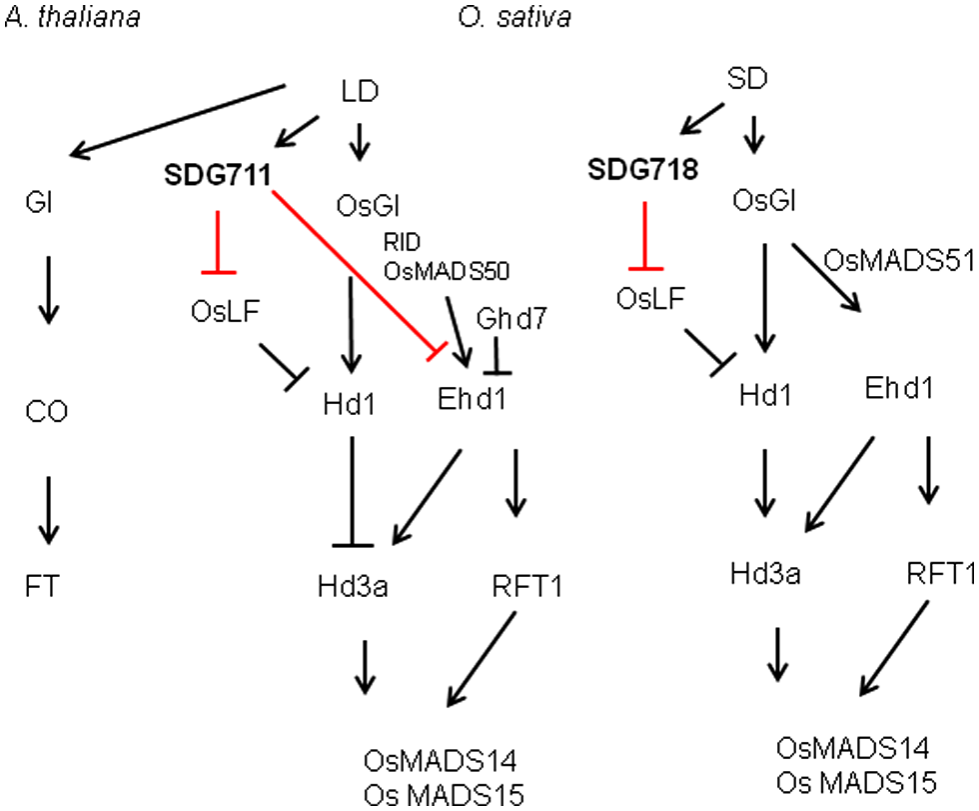
**Summary of *SDG711* and *SDG718* function in LD and SD pathways of rice flowering control compared to LD activated flowering in *Arabidopsis***.

Our data suggest that *SDG711* and *SDG718* repress *OsLF* in LD and SD, respectively, leading to the activation of *Hd1* that inhibits *Hd3a* and flowering in LD but activates *Hd3a* and *RFT1* and flowering in SD (**Figure 8**). Therefore, the two E(z) genes likely contribute to the accurate photoperiod control of flowering in rice. The increased level of *SDG711* expression in LD and that of *SDG718* in SD may be critical for *OsLF* repression. The SD-induced *SDG718* expression is consistent with the results showing that *OsVIL* genes that promote flowering in SD are also induced in SD (Wang et al., 2013). The *SDG711*-mediated LD repression of flowering is supported by previous results showing that mutants of other PRC2 genes such as *osfie2* and *osemf2b* also display an early flowering phenotype in LD (Luo et al., 2009). The observations that the *SDG711* OX only enhanced LD repression of flowering but did not affect flowering in SD (**Figure 3**), suggest that *SDG711* may be also regulated at posttranscriptional levels by day length. This hypothesis is supported by the observation that accumulation of SDG711 protein in the OX lines in LD was reduced in SD and that SDG711 binding to key flowering genes was largely reduced in SD (**Figure 7B**). This day length-dependent stability of the protein may allow SDG711 to regulate flowering only in LD. The changes of H3K27me3 levels on the *OsLF* locus in *SDG711* transgenic plants imply that PRC2-mediated repression may involve the deposition of H3K27me3 on the gene. As SDG711 was shown to be directly associated with the *OsLF* locus in LD (**Figure 7**), *OsLF* may be a primary target for rice PRC2-mediated LD-repression of flowering. The results are in agreement with recent data showing directly repression of *OsLF* by LC2 that interacts with the OsEMF2b protein (Wang et al., 2013; Yang et al., 2013). However, it is not excluded that *SDG711* or *SDG718* represses other genes that mediate *Hd1* repression.

Previous studies have shown that the flowering promoter *Ehd1* and the flowering repressor *Ghd7* could enable manipulation of slight differences in day lengths to control *Hd3a* transcription with a critical day length threshold (Itoh et al., 2010). *Ehd1* and *Ghd7* provide a gating mechanism to set critical day length for *Hd3a* expression in SD, in which *Ehd1* is repressed by the morning activation of *Ghd7* in LD (Itoh et al., 2010; Tsuji et al., 2013). The observations that *SDG711* transgenic and gain-of-function mutant plants affected the expression of *Ehd1* but not that of its upstream regulators (i.e., *Ghd7*, *MADS50* or *RID1*) and that SDG711 was associated with the *Ehd1* locus (**Figure 6**; Figures S5 and S6), indicate that SDG711 is a direct repressor of *Ehd1* in LD. In addition, H3K27me3 on the *Ehd1* locus was modulated by the expression level of *SDG711* (**Figure 6A**). Therefore, besides *Ghd7* and *OsMADS50*, *SDG711* represents an additional LD repressor of *Ehd1* (**Figure 8**). The observations that *Ehd1* expression was not clearly affected by down-regulation of *SDG718* and *SDG711* or up-regulation of *SDG711* in SD (**Figures 4B,C**), suggest that PRC2 may mainly target to the OsLF-Hd1 pathway instead of that of *Ehd1* in SD. Alternatively, the SD-induced expression of *Ehd1* (mainly due to the repression of *Ghd7* in SD) may be overwhelming, which may mask the effect of *SDG718* RNAi. In addition, the chromatin analysis data indicated that key flowering genes displayed different histone modification patterns (**Figure 6**). The changes of H3K27me3 levels on the marked genes caused by *SDG711* RNAi and OX and *SDG718* RNAi, which are correlated with their expression change in the transgenic plants in LD or SD, suggest that *SDG711*/*SDG718* may be also involved in the deposition of the mark on these loci. Collectively, the data demonstrating that the *SDG711* and *SDG718* are involved respectively, in the LD and SD signaling to promote LD repression and SD activation of flowering, suggest the involvement of PRC2 in the accurate photoperiod control of flowering in rice.

*SDG711* and *SDG718* are closely related to *Arabidopsis CLF* and *SWN*, respectively. It is suggested that *CLF* and *SWN* act redundantly to regulate vegetative growth. No vegetative phenotype observed in *SDG711* and *SDG718* transgenic plants support the idea that the two rice genes may also have a redundant function in the vegetative growth. *Arabidopsis* genome contains a third E(z) gene, *MEA*, which is mainly involved in the regulation of gene imprinting and embryo and endosperm development. The counterpart of *MEA* is not found in rice. Therefore the question arises as that whether either *SDG711* or *SDG718* plays a role in reproductive development. Our data showing that besides flowering time, *SDG711* RNAi and OX affects stamen number, pollen viability, and fertility (Figure S2) suggests that this E(z) gene may be involved in reproductive development in addition to flowering time control in rice.

## AUTHOR CONTRIBUTIONS

Xiaoyun Liu and Dao-Xiu Zhou designed the experiments and wrote the manuscript. Xiaoyun Liu, Chao Zhou, and Yu Zhao performed the experiments. Shaoli Zhou performed some images processing. Wentao Wang performed some materials cultivation. All authors have read and approved the manuscript.

## Conflict of Interest Statement

The authors declare that the research was conducted in the absence of any commercial or financial relationships that could be construed as a potential conflict of interest.
