# Supplementary material for: The rice enhancer of zeste [E(z)] genes SDG711 and SDG718 are respectively involved in long day and short day signaling to mediate the accurate photoperiod control of flowering time
Source: Front Plant Sci. 2014 Oct 31;5:591. doi: 10.3389/fpls.2014.00591 (PMC4215622; doi:10.3389/fpls.2014.00591)
Supplement: Supplementary file 1 [file Data_Sheet_1.PDF]

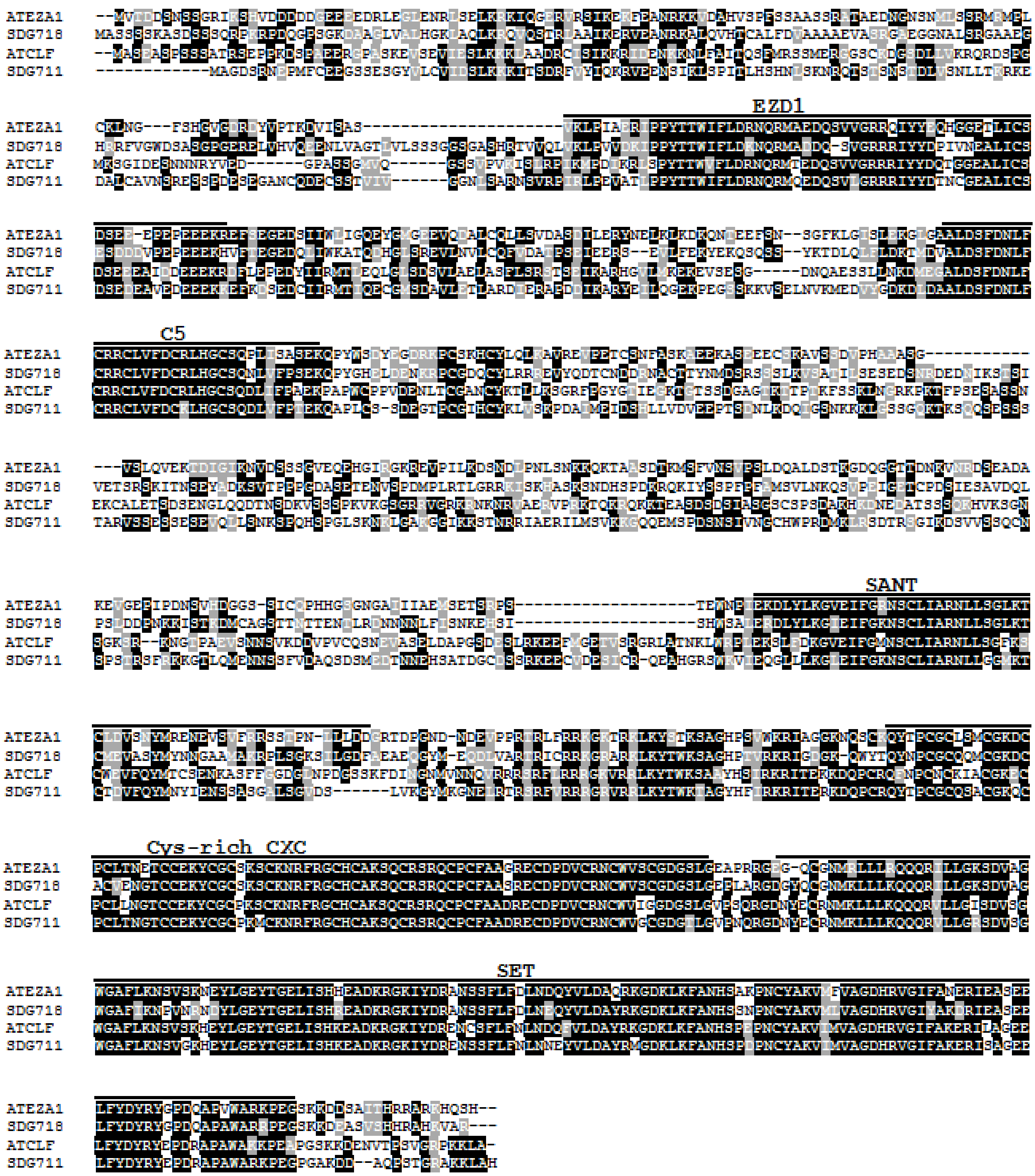

Figure S1. Amino acid sequence alignment of SDG711, SDG718 with Arabidopsis CLF and SWN. Conserved domains and motifs are over lined.

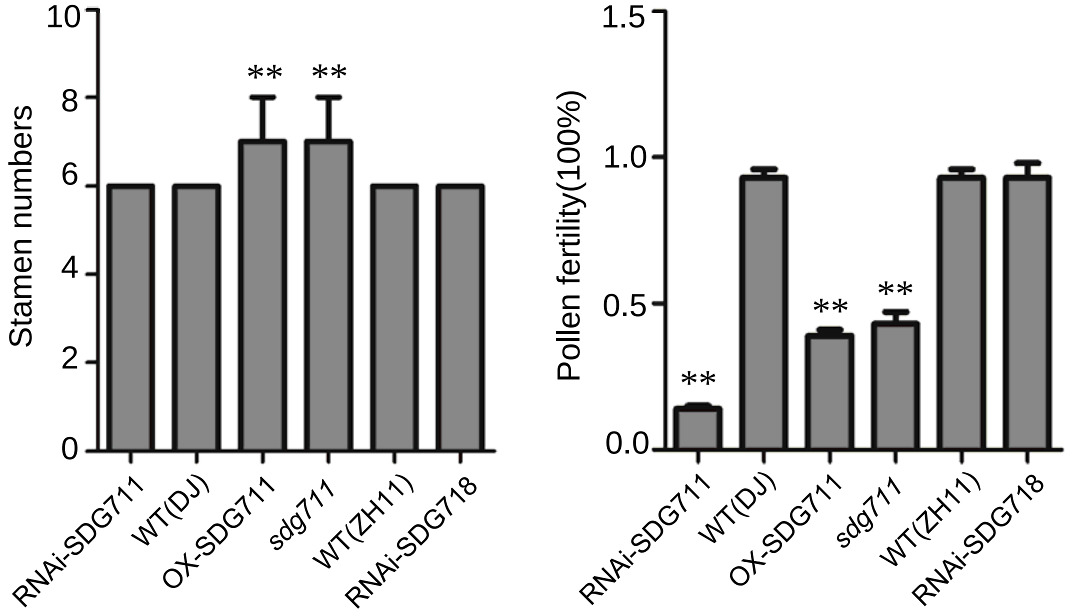

Figure S2. Comparison of stamen numbers and pollen fertility between wild type and *SDG711* and *SDG718* transgenic plants. Over-expression (OX), RNAi, and a gain-of-function T-DNA mutant (*sdg711*) plants were analyzed . Bars=means +/-SD from 10 samples. Significance of differences between wild type and transgenic plants are statistically calculated by Student's t-test (**P <0.005，*P<0.01).

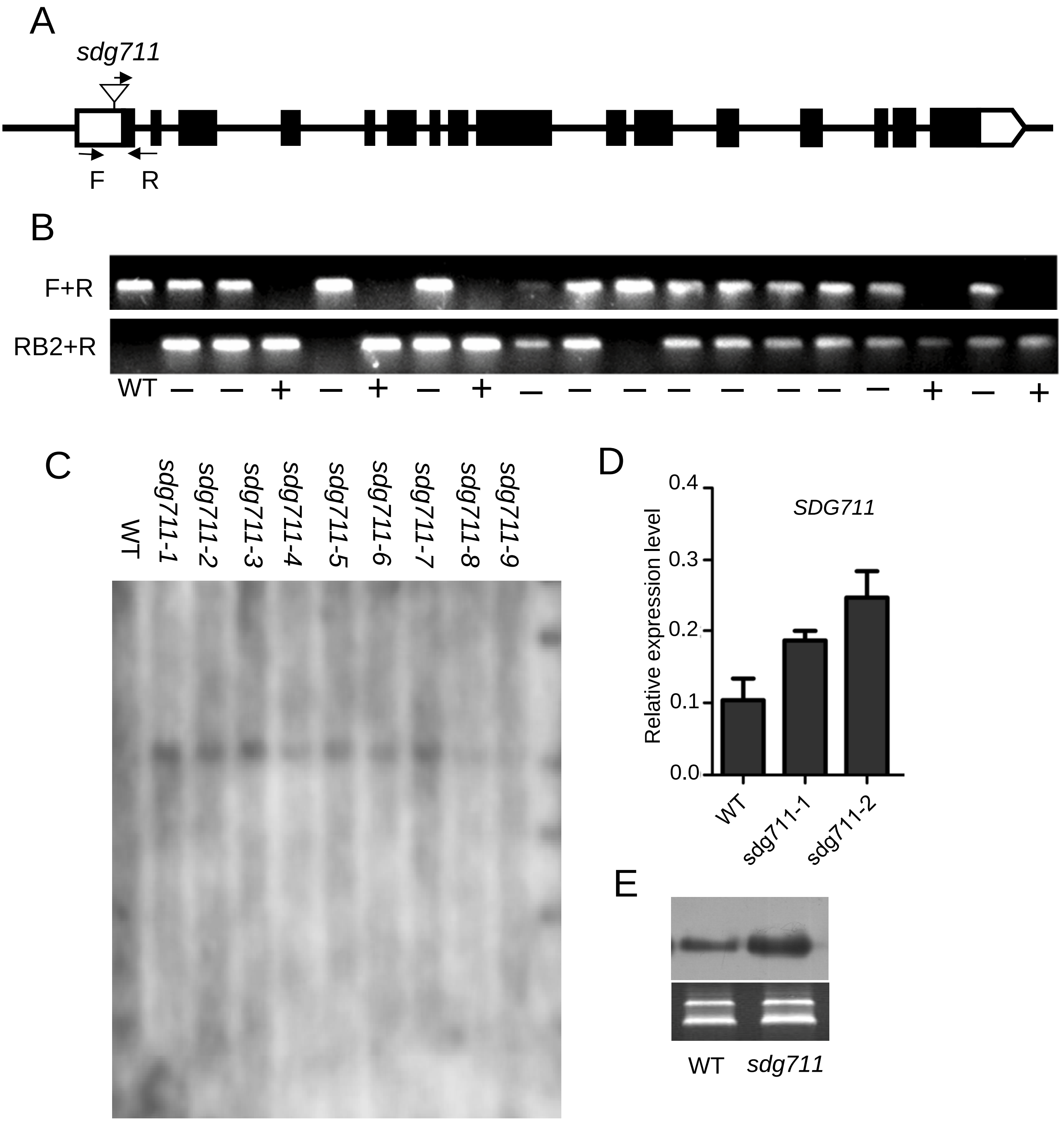

Figure S3. Characterization of a gain-of-function T-DNA insertion mutant line. **A**. Schematic representation of the *SDG711* gene. T-DNA insertion in the 5' untranslated region is indicated by an open arrow. Position of the forward (F) and reverse (R) primers used for genotyping are indicated. **B**. Identification of homozygous insertion plants by genotyping using the F and R and the T-DNA (RB2) primers. **C**. Southern blot detection of a single insertion in the genome. **D**. Relative expression levels of *SDG711* in the mutant plants compared to wild type. Bars=means +/-SD from three technical repeats **E**. Northern blotting analysis detection of full length transcripts of *SDG711* in the mutant plants.

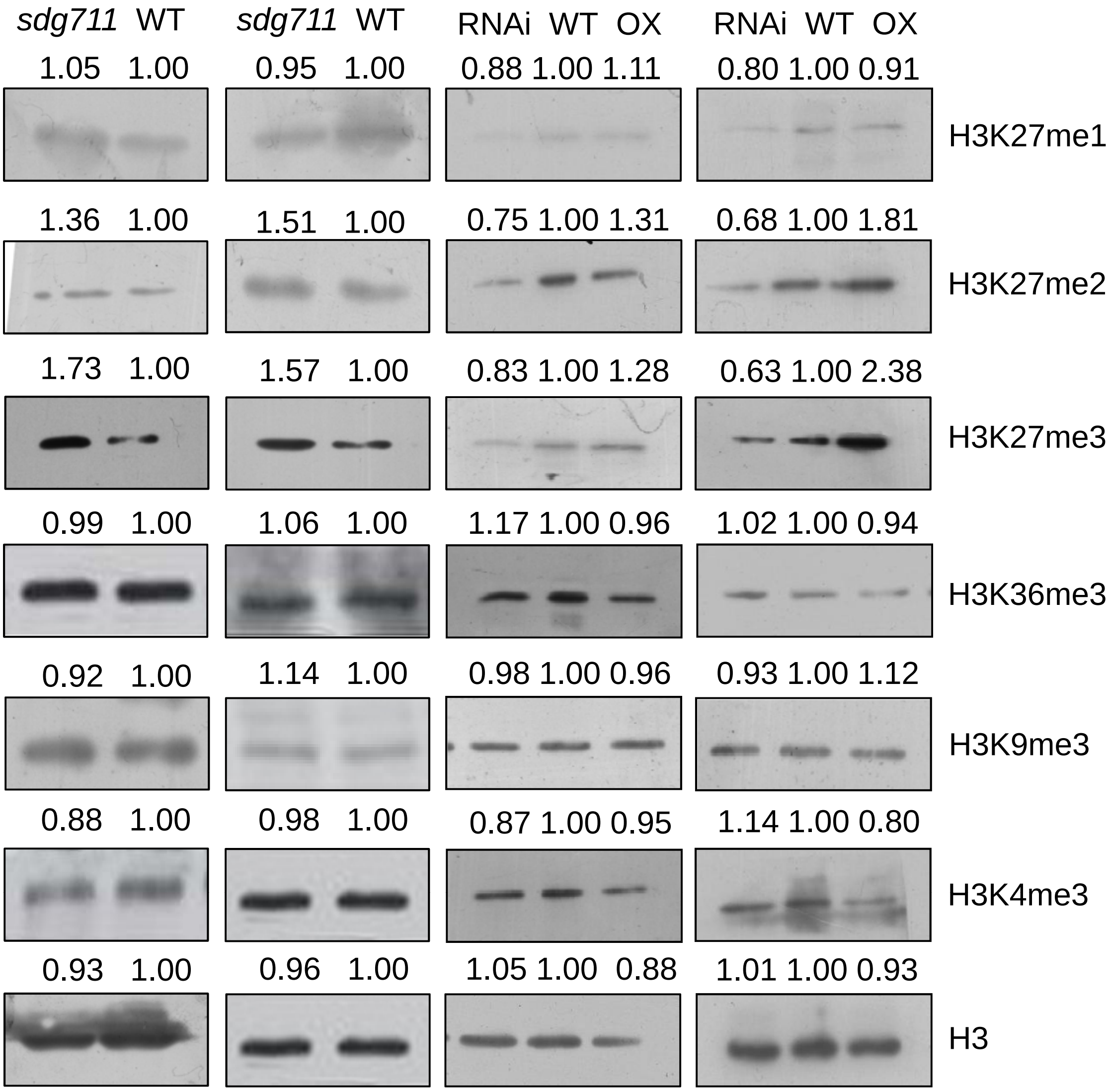

Figure S4. *SDG711* is required for overall histone H3K27me2/3. Western blotting analysis of histone modification change in *SDG711* RNAi, over-expression (OX) and the gain-of-function mutant (*sdg711*) plants (leaves) compared to wild type (WT). Antibodies against the specific histone modification modules are shown on the right. Signals relative to wild type (set as 1) are indicated on the top of the blots. At least three repeats were performed, two replicates are shown.

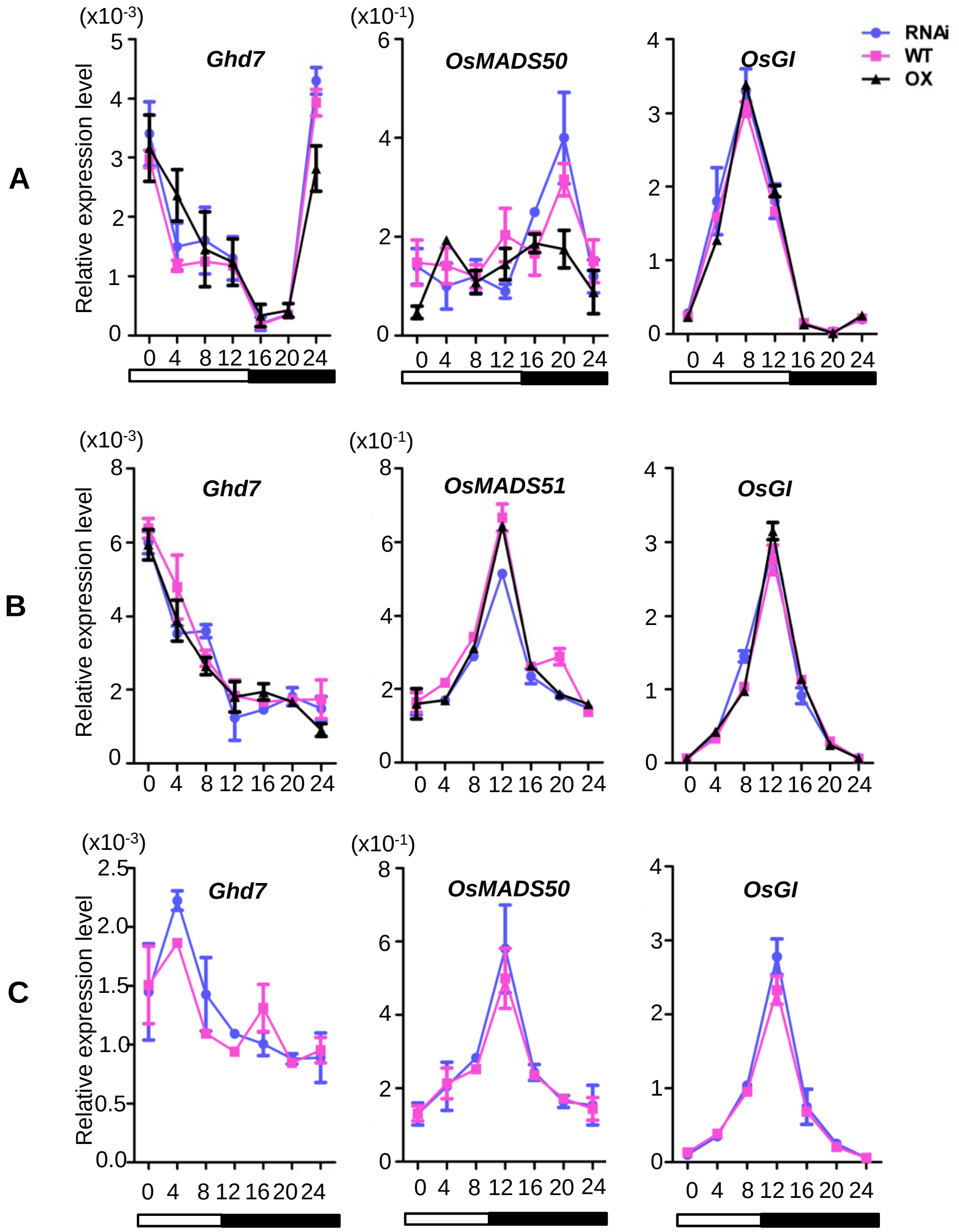

Figure S5. The expression of *Ghd7*, *OsMADS50*, and *OsGI* was not affected by *SDG711* and *SDG718* transgenes. **A**. Comparison of transcript levels of *Ghd7*, *OsMADS50* and *OsGI* in *SDG711* RNAi and overexpression plants grown in LD. **B**. Comparison of transcript levels of *Ghd7*, *OsMADS51* and *OsGI* in *SDG711* RNAi and overexpression plants with wild type grown in SD. **C**. Comparison of transcript levels of *Ghd7*, *OsMADS50* and *OsGI* in *SDG718* RNAi plants with wild type grown in LD. Bars=means +/-SD from three biological repeats.

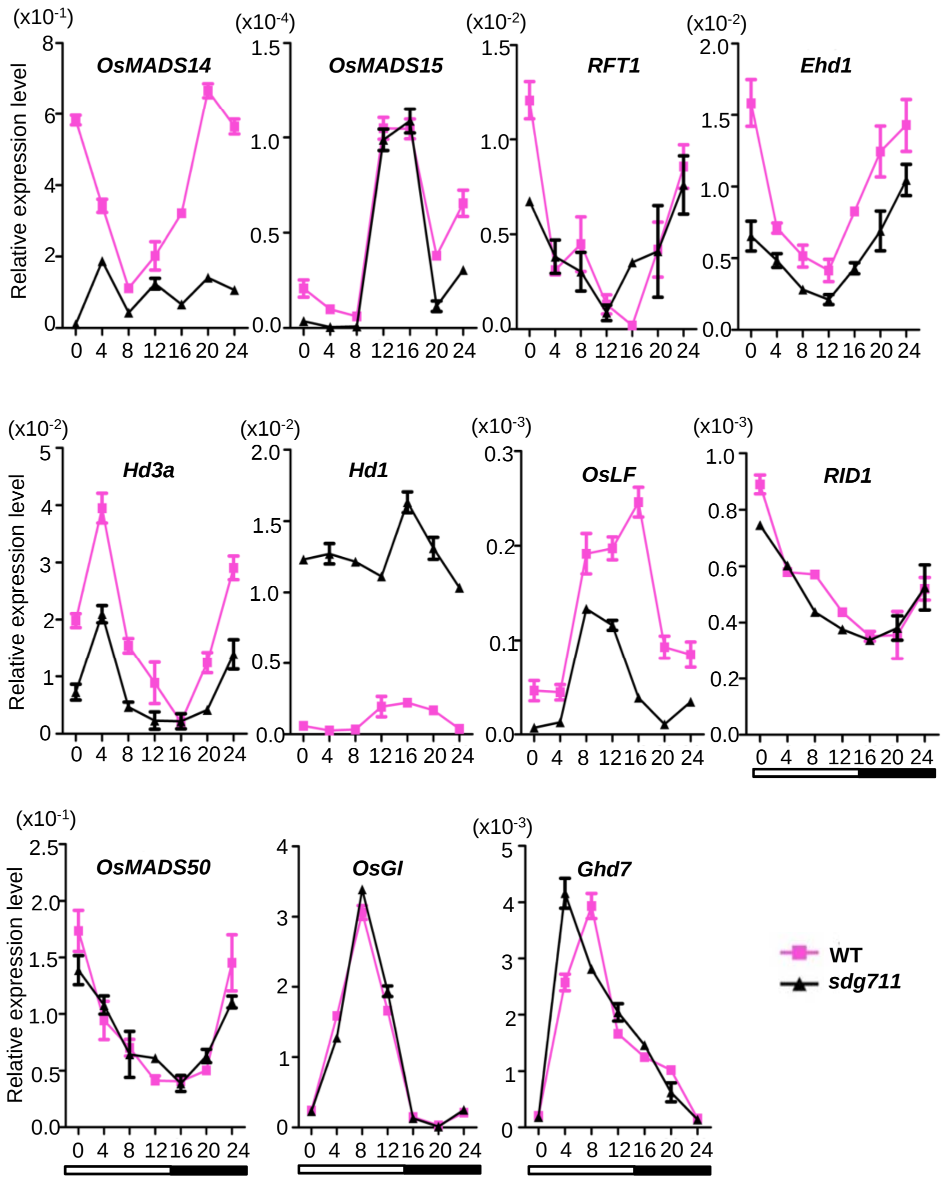

Figure S6. Analysis of flowering gene expression in wild type and the gain-of-function mutant in LD. Bars=means +/-SD from three biological repeats.

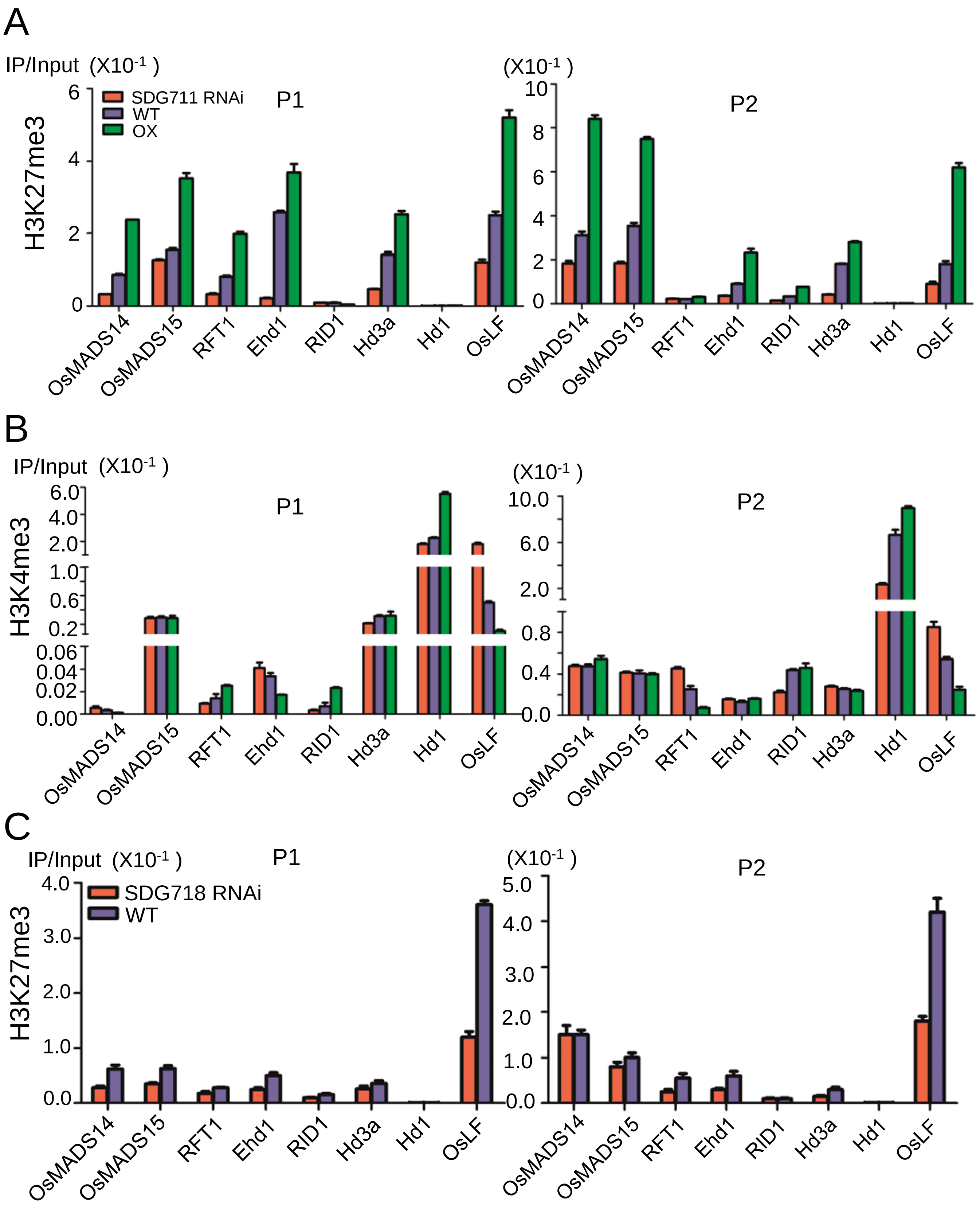

Figure S7. A biological repeat of data presented in Figure 6

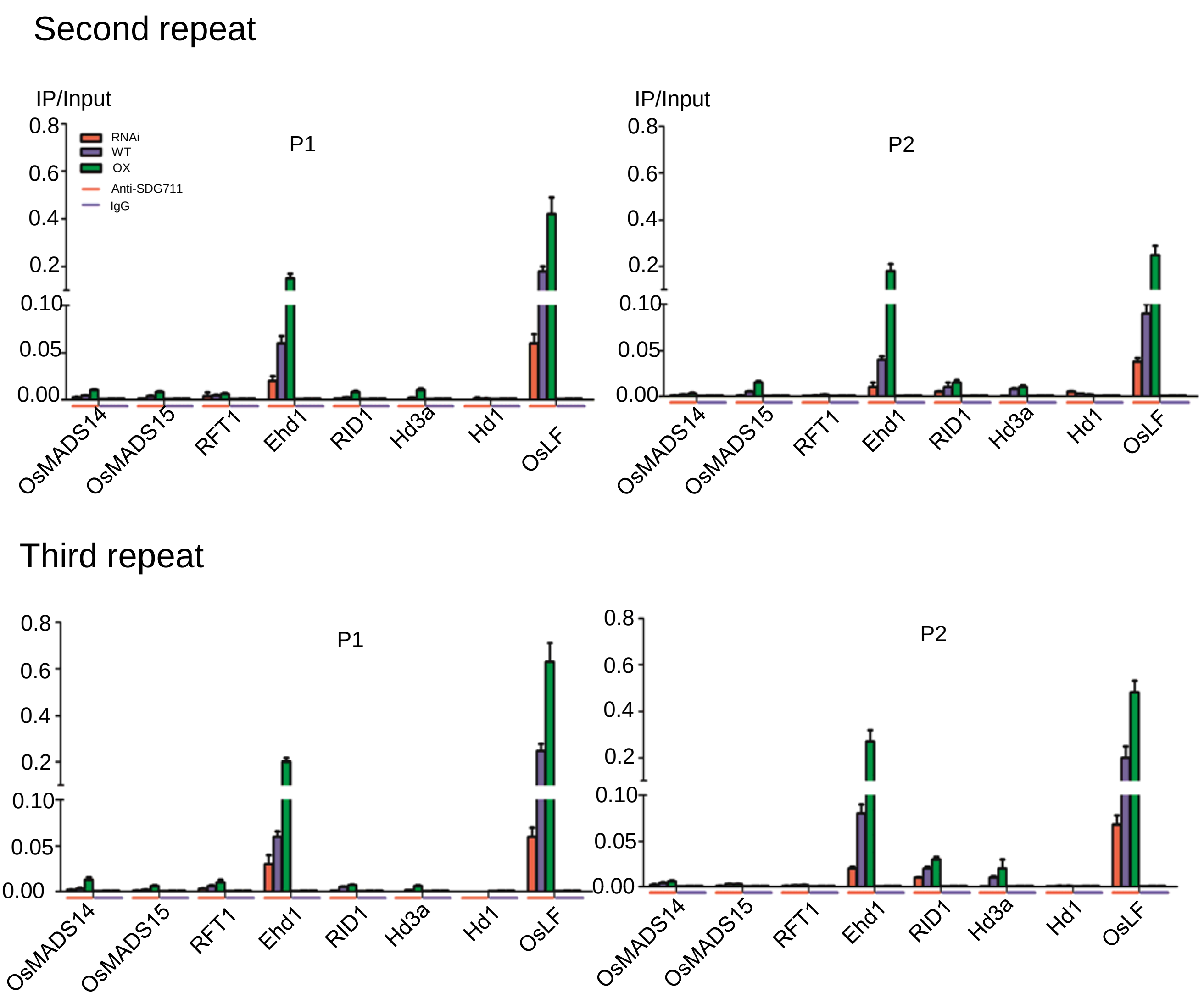

Figure S8. Two biological repeats of SDG711 ChIP experiments presented in Figure 7A.

Table S1. Nucleotide sequences of primers used in this study.

**Primers for vector construction**	
OX-SDG711-F	TTACGAACGATAGCCGGTACCTGATGGCTGGCGATTCC
OX-SDG711-R	TTTGTAATCGGATCCGGTACCGAATGGCAGGAAAGTTTTCC
Ri-SDG711-F	AGAACTAGTGGTACCAGAAGCTGACAAGCGTGGAAAG
Ri-SDG711-R	AGAGAGCTCGGATCCCGACAGGTGCAGCAGTTACAG
Pro-SDG711-F	AGA GGATCC CGAATGGCAGGAAAGTTTTCC
Pro-SDG711-R	AGA CATATG CTGATGGCTGGCGATTCC
Ri-SDG718-F	AGAACTAGTGGTACCTTGGTATTCCCTAGCGAGAAGC
Ri-SDG718-R	AGAGAGCTCGGATCCACTCCAATGAGAAATAGAGTGCTCC
Insite-SDG711-F	ACAGATTTAGTCTCGAATTTGCTCA
Insite-SDG711-R	GTTTCTCCCCGTAGGATCTCAT
Insite-SDG718-F	ATCTGTCTCTCACCACCGAGC
Insite-SDG718-R	CCTAATATTGTACTCCCTCTGTTCC
**Primers for T-DNA detection**	
Sdg711-RB2	GGACCTGCATATAACCTGCA
Sdg711- F	GCCTTCCGCCCTCCT
Sdg711-R	CGGTCCGATGTGATTTTCTT
**Primers for quantitative RT-PCR**	
Actin-QF	TGAAGATCAAGGTGGTGGCAC
Actin-QR	TGCTGGACCCGACTCATCATA
711-QF	AGGAGCCAACATCGGACAAT
711-QR	TGAGGAAACCCTTGCAGTTGA
718-QF	TCCCTAGCGAGAAGCAACCAT
718-QR	GCATTTCGGTCATCATTGCAC
OsLF-QF	AACCCTAGGGAATGGCAATG
OsLF-QR	CGCCCAAATGCAAGTACAGT
OsMADS14-QF	GCCAACTAATGCTCGAGTCC
OsMADS14-QR	CAGCTGCTGCAGGGTAGTTA
OsMADS15-QF	CGTCGTCGGCCAAACAG
OsMADS15-QR	TGACTTCAATTCATTCAAGGTTGCT
OsMADS50-QF	CAGGCCAGGAATAAGCTGGAT
OsMADS50-QR	TTAGGATGGTTTGGTGTCATTGC
OsMADS51-QF	GTTTGCTCTGCTCCTACTC
OsMADS51-QR	ACTCCTCCTCCAGCATTGAA
RFT1-QF	TGGTGTTCGTGCTGTTCCA
RFT1-QR	TTGTAGAGCTCGGCGAAGTTC
Hd1-QF	TCAGCAACAGCATATCTTTCTCATCA
Hd1-QR	TCTGGAATTTGGCATATCTATCACC
Hd3a-QF	GCTCACTATCATCATCCAGCATG
Hd3a-QR	CCTTGCTCAGCTATTTAATTGCATAA
Ehd1-QF	GGATGCAAGGAAATCATGGA
Ehd1-QR	AATCCCATCGGAAATCTTGG
RID1-QF	CGACGACAATAGCTCGATCGC
RID1-QR	GTGCATGGTCACGGAGCCTT
OsGI-QF	TGGAGAAAGGTTGTGGATGC
OsGI-QR	GATAGACGGCACTTCAGCAGAT
Ghd7-QF	AAATCCGGTACGCGTCCAG
Ghd7-QR	GACATAGGTGGATGGCGGTG
**Primers for ChIP analysis**	
Actin-CF	TGCGTCAGGAATTCAGAACCA
Actin-CR	AGCACCACGAACCTTGACCAT
OsLF-CF1	CAGCATTTTTGGTTGGGAGT
OsLF-CR1	ACCCGAAGGCGTCCATGT
OsLF-CF2	GAGCTTCGAGCTCCACTCC
OsLF-CR2	AACGCGAACCTATCGAGGA

OsMADS14-CF1	CGCTCCCTTTAAAATCCCC
OsMADS14-CR1	AATTAACCCTACCCACCTCGC
OsMADS14-CF2	ACAAGATCAACCGGCAGGTGA
OsMADS14-CR2	TGCCCTTGGTGGAGAAGATGA
OsMADS15-CF1	TCCTTCTCACGTAACATGCCG
OsMADS15-CR1	ATGCTGTCGAGCTGTGATTGG
OsMADS15-CF2	TTGCCATTTCTGGAGCACC
OsMADS15-CR2	CAGCAGCTGAAATCCATGGA
RFT-CF1	TGGATTGAACGGCAGGAGA
RFT-CR1	GCCGGAAAAACTCTAGGCATT
RFT-CF2	TCGTCCGGATCACTAACCTCA
RFT-CR2	ACGTCCTCATGTCATTGCCAC
Ehd1-CF1	TGACGAGCCATCCCTTTGAA
Ehd1-CR1	AGCCCATTGTGACGGCATAT
Ehd1-CF2	AATCTCGAAAAACCCGGTCA
Ehd1-CR2	CATTTTCTAGAGGCGCTAGCG
Hd1-CF1	ACAAGAGCCATGCGAGGTAGA
Hd1-CR1	GCTTGCACCACATGGACAAGT
Hd1-CF2	AGCAGCAACAACGGCATGTATT
Hd1-CR2	CCGATCTTGGTTGTTTTCGATG
Hd3a-CF1	CACGTACAGGAAGACGATGCA
Hd3a-CR1	TGGATCGAGCTGTGGTTGAGA
Hd3a-CF2	TCAAGGTCACCTATGGCTCCA
Hd3a-CR2	TGCACCAACTACGACACATGG
RID1-CF1	CGCGATATGATTGCATCATCG
RID1-CR1	TTCTGCAGGTTGCAGTTGCTG
RID1-CF2	CGAAACTAGTGCCAATGCCAC
RID1-CR2	TTGGCATCCAGAGTCGCAA
